# Prevalence of Suicide

**Published:** 1889-07

**Authors:** W. H. Walsh


### PREVALENCE OF SUICIDE.

The terrible increase in the number of suicides, as well as the social significance of voluntary death, making it one of the great evils of modern civilization, demands attention.

It is doubtful if any authority on the subject, as yet enveloped in great metaphysical obscurity, could have more weight, or be more correct in premises than that of the expert physicians whose knowledge of and constant experience in mental diseases have made their judgment of suicide, or rather of suicidal tendency, the very best.

Among this class of experts in Massachusetts, it is doubtful if there are any more prominent than Dr. Colles, the superintendent of the McLean Asylum, at Somerville; Dr. Fisher, superintendent, and Dr. Dewey, his assistant at the South Boston Insane Asylum; Dr. Folsom and Dr. Jelly, of that city.

Every one of these experts agree in the main that suicide is purely a mental rather than a physical disorder, though it may be the result of a lowering and starving of the physical resources; and that primarily it is an effect of the struggle for human existence and of human selection.

The cure declared in all their philosophic testimony is contained in this one precept: To preserve and develop a healthy physical organism, and engender well ordered and energetic sentiments in the mental and moral character and mental faculties.

"The first and predominating cause," said Dr. Colles, "whose sequence is suicide, is a morbid sentiment, a morbid condition of the mind, disordered passions and delirium of the understanding. The physical condition, of course, has much to do with it, for a weak physical condition induces worry and morbid excitement of the mental organs. The result is simply a progressive sequence from the physical to the mental disorder. Melancholia, in every case, is the predominating characteristic. Here in the McLean Asylum, out of one hundred and sixty-four patients, thirty-four are afflicted with melancholia, every one of whom shows a tendency to suicide, and requires constant surveillance.

It is a tradition of this asylum, and I have no doubt the tradition is a true one, that in proportion to the number of patients we have more here possessed of a suicidal tendency than in any other asylum in this country. Some authorities agree that a man may have melancholia without any essential intellectual disorder, and that he may think as calmly, intelligently and dispassionately of a means for committing suicide as though there existed no disorder whatever of his mental faculties. In contrast to that, we have madness where mental disorder begins with disorder of the ideas.

Still another form of mental trouble is the disorder of ideation, or fixed ideas. It is this last which induces some people, after climbing to a high place, to jump off, and the impulse, with them, is irresistible. But this type of suicide, if it can be called such, is entirely different from that which I am considering as the result of melancholia. A person may be in the best of spirits and in the soundest condition every way, and yet be subject to ideation. There are thousands of such people in this country and in every other, and no one thinks for a moment of questioning their sanity.

Have you ever had the blues? That very feeling in mental distress is the first step in melancholia, the sequence of which, broadened and deepened so that the whole mind feels a pervading gloom, is suicide. I could point out a dozen cases of melancholia in this asylum where the patients are perfectly rational and will talk intelligently of their mental disorder.

The usual characteristic is that of feeling utterly discouraged, an utter depression of the sensitive faculties, which may and does result from a variety of causes, and for which the patient feels the only cure is suicide.

It is the same in the world at large. The more enlightened the intellect the more reasonable does this conclusion seem, and the more readily does the mind decide upon a means for taking away life.

One reason why the ignorant classes are the least given to suicide is that they do not reason readily, and are more superstitious. The more enlightened one becomes the less one fears to die. I can scarcely consider it possible how any person may be sane and commit suicide, for the act itself is presumptive evidence, to my mind, of insanity. The basis of melancholia, of course, is disorder of physical and mental feeling. As a rule, the intellect in melancholia, retains, in great degree, its integrity, but, as the gloom and despondency become more and more profound, the victim becomes absolutely hopeless of the future; his thoughts dwell upon the past, and he reasons out a suicidal plan as the only remedy.

The belief which grows out of this rersoning is more or less logical, and as a logical conclusion from this state of despair it seems best to make an end of it, and terminate a hopeless condition.

As the enlightened mind works more promptly and more logically than the ignorant in its morbid tendency to suicide, so too, it is the more susceptible to reason and to curative influences. Melancholia is curable, and in many cases where a patient manifests a decided tendency to suicide, all that is required is a restoration to health physically, and as the physical condition improves, the mentality partakes of the benefits and is built up and restored accordingly."

The moral to be drawn from all this expert testimony is that suicide is a malady of cultivation, and that melancholia, a sinking of the physical and mental organisms, is the primal and predisposing cause. The cause and cure for suicide is in the economical and social conditions of civilization. The evolution of the intellectual faculties and the egoistical principle of self-love and self-preservation lie at the root of this great moral and social fact. Practically considered, the victim to a morbid sentiment is unhealthy in body and in mind, and that remedy exists in the development of a healthy and well-ordered condition of both.

W. H. WALSH.

